# *Ficus carica* and *Sizigium cumini* Regulate Glucose and Lipid Parameters in High-Fat Diet and Streptozocin-Induced Rats

**DOI:** 10.1155/2020/6745873

**Published:** 2020-10-29

**Authors:** El-Shaimaa A. Arafa, Waseem Hassan, Ghulam Murtaza, Manal Ali Buabeid

**Affiliations:** ^1^College of Pharmacy and Health Sciences, Ajman University, Ajman 346, UAE; ^2^Department of Pharmacology and Toxicology, Faculty of Pharmacy, Benisuef University, Beni-suef, 62514, Egypt; ^3^Department of Pharmacy, COMSATS University Islamabad, Lahore Campus, Lahore 54000, Pakistan

## Abstract

Obesity linked diabetes, popularly known as diabesity, has been viewed as a direct product of the modern lifestyle in both developed and developing countries, and its increased prevalence is seen as a major threat to public health globally. *Ficus carica* (FC) and *Syzigium cumini* (SC) are part of indigenous flora with traditional medicinal properties. Fresh seeds of SC fruit and fruit of FC were collected and macerated to obtain the final extract. Wistar rats were divided into seven groups fed either on a normal diet or high-fat diet (HFD) along with streptozocin (STZ) to induce diabesity. The crude extract of FC (FC.Cr.) and SC (SC.Cr.) were administered at 250 mg/kg/day and 500 mg/kg/day in induced diabesity state. Body weights, blood glucose level, complete blood count (CBC), cholesterol, triglycerides (TG), low-density lipoprotein (LDL), very-low-density lipoprotein (VLDL), and high-density lipoprotein (HDL) were recorded to analyze their effects on glucose and lipid metabolism. Further, superoxide dismutase (SOD) and malondialdehyde (MDA) were measured to examine their effects on lipid peroxidation and ant oxidative enzyme. Results showed that both FC.Cr. and SC.Cr. have the potential to control obesity-linked type 2 diabetes mellitus (T2DM) by lowering the body weights, serum glucose, cholesterol, TG, LDL, and VLDL, while increasing the protective effects of HDL dose-dependently. The crude extract of both plants showed significant activity to raise SOD and curb MDA under diabetic states. It was concluded that both FC.Cr. and SC.Cr. exhibited remarkable therapeutics potential in HFD-STZ-induced diabetic rats. However, we found that the effects of SC.Cr. are relatively more pronounced as compared to FC.Cr. in almost all parameters.

## 1. Introduction

The merger of obesity and diabetes led to the term “diabesity.” Obesity causes gradual defects in insulin discharge along with increased insulin resistance (hyperinsulinemeia) which has the capacity to cause diabetes [1]. Obesity and type 2 diabetes mellitus (T2DM) are strongly interlinked and have a similar kind of pathophysiology [2]. The occurrence of obesity has reached the epidemic level all over the world. It is threatening globally, and many countries are facing the problem in its treatment, clinical management, and prevention [3]. Class II and class III obesity is directly linked with great danger to having diabetes. Women with obesity are at 10 times risk while obese men have 11.2 times more risk of developing diabetes [4]. The increased concurrence of obesity and diabetes compelled the World Health Organization (WHO) to state it as the “21st century epidemic.” Furthermore, epidemiological studies have revealed that approximately 60-90% of patients with T2DM have been obese or overweight. It is potentially a predisposing factor in the late development of T2DM. Several research and studies proved that obesity is a robust risk factor to develop T2DM [5].

Interest in herbal therapies is increasing with the passage of time primarily due to the side effects of current therapeutic agents (oral hypoglycemic agents and insulin) used in diabetes mellitus. Many indigenous medicinal plants are useful to treat diabetes. *Ficus carica* Linn. (FC) is locally known as Anjeer or fig belongs to the family *Moraceae* [6]. It is generally found in tropical and subtropical regions of the world including the Indian subcontinent, southwest Asia, and Mediterranean regions. It is a plant with cultural and historic background, and its name is mentioned in traditional systems of medicine like Ayurveda, Unani, and Siddha [7, 8]. Its leaves, bark, tender shoots, fruits, seeds, and latex are used in traditional remedies such as antispasmodic, antipyretic, anthelmintic, antimutagenic, antioxidant, antifungal, hepatoprotective, and hypoglycemic agents [6, 9]. In the context of this study, its hypolipidemic and hypoglycemic activities are reported in various diabetes-induced models [9–11]. The activities of FC are also reported in metabolic syndrome and related disorders [12]. Moreover, the protective effects of the FC leave on glucose and lipid metabolism along with the cytoprotective effects on pancreatic *β*-cells are described in the literature [13]. Badgujar et al. has prospectively termed FC as a potential pharmaceutical candidate which can be effective against anemia, cancer, diabetes, leprosy, liver diseases, paralysis, skin diseases, and ulcers [7]. In addition, its activities as anticancer [14], antimicrobial [15], and antiwarts agent [16] can also be found in the scientific literature. Its in vitro antiviral potential activity is described against herpes simplex type 1 (HSV-1), echovirus type 11 (ECV-11), and adenovirus (ADV). Authors argued that the hexanic and hexane-ethyl acetate extracts inhibited the multiplication of viruses at concentrations of 78 mg/mL [17, 18].

*Syzygium cumini* (SC) is an eatable, commonly known as black plum which belongs to the family *Myrtaceae*. It contains a wide array of active biological constituents like gallic acid, anthocyanins, tannins, vitamin C includes cyanidin, petunidin, and malvi-din-glucoside among other substances [19]. It is commonly being widely used to treat diabetes by traditional practitioners over many centuries [20]. Its ripe fruits are used to prepare jams, jellies, squashes, and wine [21]. Studies have proved that methanol and ethyl acetate extracts of SC are effective in STZ-induced diabetes [22]. Further, its polyphenol-rich extract has shown pharmacological properties in restoring glucose tolerance and inducing insulin secretion [23]. In addition to antidiabetic properties, its pharmacological potential as antioxidative [24], antipyretic [25], anti-inflammatory [26], anticancer [27], antibacterial [27], and psychopharmacological [28] agent are documented in modern literature.

Thus, the aim of this study was to evaluate the antidiabetic, hypolipedmic, and antioxidant potentials of *FC* and *SC* in high-fat diet (HFD) and streptozocin (STZ-) induced diabetic rats. The antidiabesity effects of these two potential plants were compared with metformin.

## 2. Materials and Methods

### 2.1. Preparation of Extract

Whole fresh fruit of SC and FC were purchased from a local herbal store and identified according to the protocols. The plants were dried and macerated with 70% aqueous methanol. The filtrates were then evaporated in a rotary evaporator as standard procedures with slight modifications [29]. Crude extracts of FC (FC.Cr.) and SC (SC.Cr.) were freeze-dried and stored for further experimentation.

### 2.2. Experimental Animals and Measurement of Physical Parameters

STZ is appropriately mixed in the citrate buffer, and the calculated volume of solution is injected intraperitoneally in designated experimental rats. STZ has a dose-dependent effect on *β*-cells; hence, its low dose of 30 mg/kg/day was used for 2 days [30] to induce T2DM like symptoms. Wistar rats weighing between 170-200 g were kept under standard environmental conditions of temperature (25 ± 2°C), relative humidity (50-55%), and 12/12 hours light and dark cycles. Animals were divided into seven groups each having six animals. The control group was fed on a normal diet, while the other six groups of rats were fed on HFD for 8 weeks. After three weeks, all groups, except the control group, were administered with a low dose of STZ for two days. Pharmacological intervention with FC.Cr. (250 mg/kg and 500 mg/kg) or SC.Cr. (250 mg/kg and 500 mg/kg) was then initiated in designated HFD/STZ-administered groups. The plasma glucose level, body weight, food intake, and urine flow were measured periodically to monitor the induction of diabesity state and considered at the end of the study duration.

### 2.3. Measurement of Fasting Blood Glucose

For the monitoring of blood glucose levels, blood samples were collected by the tail cut method following standard methods [31]. The tail is cut with the help of sharp surgical scissors, and the tail is pressed to collect the blood on the wound-tip which is directly applied on the glucometer strip and measure the glucose level. The cut tail is wrapped in water-soaked cotton plug for at least 3-4 minutes to stop the unnecessary bleeding until the animal gets recovered from anesthesia.

### 2.4. Hematological Parameters

The blood sample was collected from the animals' eyes using nonheparinized capillary tubes, and hematological parameters were performed as per the reported procedure [32]. The determined parameters include platelet count, total leukocyte count, hemoglobin, WBCs, and RBCs.

### 2.5. Serum Lipid Analysis

After four weeks of the treatment, the animals were anaesthetized with a mixture of ketamine (50 mg/kg) and xylazine (5 mg/kg) i.p. The blood samples of all the animals of different groups were collected from cardiac puncture and separated by centrifugation at 5000 rpm for fifteen minutes. Serum cholesterol, triglycerides (TG), high-density lipoprotein (HDL), very-low-density lipoprotein (VLDL), and low-density lipoprotein (LDL) were measured according to the methods recommended in respective kits [33].

### 2.6. Measurement of Serum Superoxide Dismutase (SOD)

Serum was obtained from different experimental groups as per standard operation, and the analysis of SOD was carried out by commercially available kits according to the given protocols [33]. The absorbance was measured at 505 nm for SOD. The results were then calculated according to the manufacturer's instruction and expressed as units per milliliter.

### 2.7. Determination of Lipid Peroxidation

Malondialdehyde (MDA) as a marker for lipid peroxidation was determined in serum by the double heating method of Draper and Hadley with some modifications. The principle of the method is based on the spectrophotometric measurement of the color produced during the reaction of TBA with MDA. For this purpose, 2.5 mL of 100 g/L trichloroacetic acid solution was added into 0.5 mL serum in a centrifuge tube and placed in a boiling water bath for 15 min. After cooling under tap water, the supernatant was transferred into a test tube containing 1 mL of 6.7 g/L TBA solution and placed again in a boiling water bath for 15 min. The solution was then cooled under tap water, and its absorbance was measured spectrophotometrically at 532 nm as per instructions [33]. The concentration of MDA was calculated using the following equation

MDA (nmoL/mL) = ((absorbance of sample/absorbance of standard) × 100).

### 2.8. Statistical Analysis

All results were shown as mean ± SEM. The data interpretation was done through the statistical analysis “one-way analysis of variance (ANOVA).” The results were analyzed by using the Graph Pad Prism software version 5. The significant effects were considered where *P* < 0.05.

## 3. Results

### 3.1. FC.Cr. and SC.Cr. Reverses HFD- and STZ-Induced Plasma Glucose

**Figure 1:**
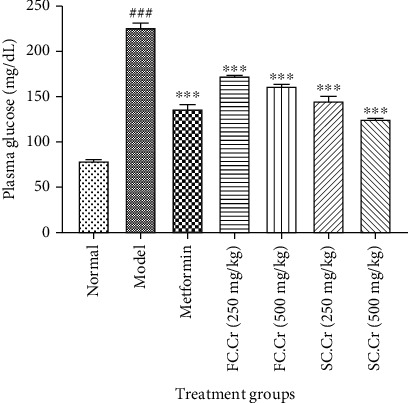
Graphical representation of fasting blood glucose level (mg/dL) after intervention with FC.Cr. and SC.Cr. in HFD-STZ-induced rats. The fasting blood glucose level of FC.Cr. and SC.Cr. at doses 250 mg/kg and 500 mg/kg is compared with the standard drug metformin at the end of the study duration. The values are expressed as the mean of six animals in a group. Statistical analysis is performed with one-way ANOVA followed by Tukey's test. All the groups are compared with the HFD-STZ group, while the HFD-STZ group is compared with the normal group. The results are considered significant (^∗^) if*P* < 0.05and (^∗∗^) if*P* < 0.01and highly significant (^∗∗∗^) if*P* < 0.001.

The level of plasma glucose remains a key prognostic factor in the diagnosis of diabetes. Our results manifested that the administration of STZ along with HFD robustly increased the fasting plasma glucose level, and the trend continued till the end of the study duration. The intervention by FC.Cr. showed a significant dose-dependent reduction in plasma glucose levels as it was recorded at 165 ± 5 mg/dL at 250 mg/kg and 150 ± 5 mg/dL at 500 mg/kg. Similarly, the plasma glucose levels were 140 ± 5 mg/dL and 120 ± 5 mg/dL at 250 mg/kg and 500 mg/kg of SC.Cr., respectively. It was interesting to notice that the effects of SC.Cr. were more pronounced in controlling plasma glucose as compared to FC.Cr. (Figure 1).

### 3.2. Effects of FC.Cr. and SC.Cr. on Body Weights

**Figure 2:**
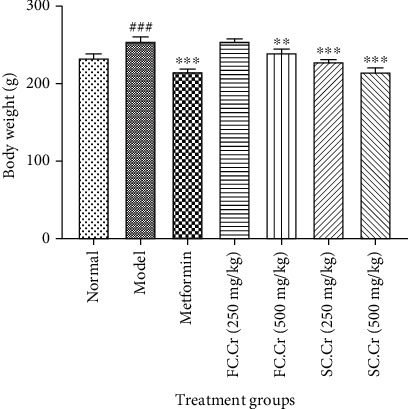
Effects of FC.Cr. and SC.Cr. on body weights in HFD-STZ-induced rats with established diabesity. The rats were induced with HFD-STZ to mimic diabesity. FC.Cr. and SC.Cr. were administered at 250 mg/kg and 500 mg/kg after three weeks till the end of the study duration. Body weights were measured on a daily basis, and the final weights were compared with the standard drug metformin. The values are expressed as the mean of six animals in a group. Statistical analysis is performed with one-way ANOVA followed by Tukey's test. All the groups are compared with the HFD-STZ group, while the HFD-STZ group is compared with the normal group. The results are considered significant (^∗^) if*P* < 0.05(^∗∗^) and if*P* < 0.01and highly significant (^∗∗∗^) if*P* < 0.001.

A relatively notable increase in the body weights of animals fed on STZ along with HFD was recorded. It was noted that the increase in the body weights was not overwhelming in terms of grams as was recorded by other studies feeding animals on HFD alone [34]. The effects of FC.Cr. on body weights were unremarkable, especially at 250 mg/kg, but SC.Cr. at both low (250 mg/kg) and high dose (500 mg/kg) was able to decrease the body weight to some extent. Metformin was used as a standard drug, which is well known to reduce body weights [35]. Interestingly, the ability of SC.Cr. at a higher dose to reduce the body weights was similar to that of metformin (Figure 2).

### 3.3. Effects of FC.Cr. and SC.Cr. on Complete Blood Count (CBC)

Blood was collected in test tubes already containing EDTA to avoid the coagulation of blood. The CBC results are tabulated in Table 1. Red blood cells, white blood cells, platelets, and hemoglobin are measured. There was no noticeable change recorded on hemoglobin, RBC, and platelet count; however, expectedly, a significant increase was observed in WBC count as obesity is linked with the low-grade inflammation [36, 37]. FC.Cr. at 250 mg/kg reduced the WBs to 8.9 ± 0.66 (10^3^/UL) and to 8.1 ± 0.53 (10^3^/UL) at 500 mg/kg. Similarly, SC.Cr. decreased the WBs to 11 ± 0.34 (10^3^/UL) and 8.0 ± 0.38 (10^3^/UL) at 250 mg/kg and 500 mg/kg, respectively, as compared to the HFD-STZ group which was recorded at 13 ± 0.21 (10^3^/UL).

### 3.4. FC.Cr. and SC.Cr. Improves the Plasma Lipid Profile in Diabesity

**Figure 3:**
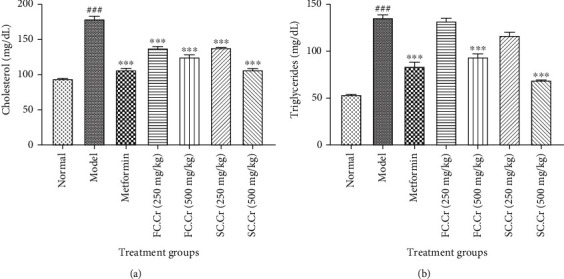
Effects of FC.Cr. and SC.Cr. on plasma levels of cholesterol (mg/dL) and TG (mg/dL) in HFD-STZ-induced rats. FC.Cr. and SC.Cr. were administered at 250 mg/kg and 500 mg/kg after three weeks till the end of study duration. Cholesterol (a) and TG (b) were measured after drawing plasma from the treated and normal group of rats. The values are expressed as the mean of six animals in a group. Statistical analysis is performed with one-way ANOVA followed by Tukey's test. All the groups are compared with the HFD-STZ group, while the HFD-STZ group is compared with the normal group. Results are considered significant (^∗^) if*P* < 0.05(^∗∗^) and if*P* < 0.01and highly significant (^∗∗∗^) if*P* < 0.001.

**Figure 4:**
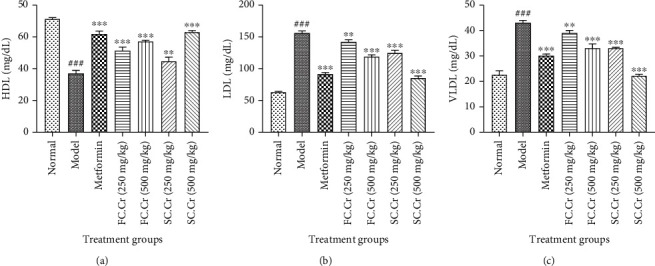
Effects of FC.Cr. and SC.Cr. on plasma levels of HDL (mg/dL), LDL (mg/dL), and VLDL (mg/dL) in HFD-STZ-induced rats. FC.Cr. and SC.Cr. were administered at 250 mg/kg and 500 mg/kg after three weeks till the end of study duration. HDL (a), LDL (b), and VLDL (c) were measured after drawing plasma from the treated and normal group of rats. The values are expressed as the mean of six animals in a group. Statistical analysis is performed with one-way ANOVA followed by Tukey's test. All the groups are compared with the HFD-STZ group, while the HFD-STZ group is compared with the normal group. The results are considered significant (^∗^) if*P* < 0.05and (^∗∗^) if*P* < 0.01and highly significant (^∗∗∗^) if*P* < 0.001.

All major parameters of serum lipid profile (TC, TG, LDL, HDL, and VLDL) were measured to analyze the lipid metabolic activities of FC.Cr. and SC.Cr. Raised levels of plasma cholesterol induced by HFD and STZ were significantly brought back by FC.Cr. and SC.Cr.; however, the relative control by SC.Cr. at 250 mg/kg (140 ± 5 mg/dL) and 500 mg/kg (130 ± 5 mg/dL) was relatively more significant as compared to FC.Cr. at 250 mg/kg (140 ± 5 mg/dL) and 500 ± 5 mg/kg (100 ± 5 mg/dL) (Figure 3(a)). A similar pattern of control was observed in the TG level, where SC.Cr. at 500 ± 5 mg/kg was found more effective as compared to FC.Cr. at similar doses (Figure 3(b)). The relative TG control difference was also obvious at 250 mg/kg (135 ± 5 mg/dL compared to 115 ± 5 mg/dL) dose of both SC.Cr. and FC.Cr. Similarly, the plasma level of HDL, which was suppressed in the HFD and STZ administered group was remarkably and dose-dependently restored by FC.Cr. and SC.Cr. Likewise, the amount of control with SC.Cr. at 250 mg/kg and 500 mg/kg was better than FC.Cr. at 250 mg/kg and 500 mg/kg doses (Figure 4(a)). Finally, the plasma levels of LDL and VLDL were decreased when FC.Cr. and SC.Cr. were administered in the presence of HFD and STZ. The LDL and VLDL levels at the doses of 250 mg/kg and 500 mg/kg of FC.Cr. and SC.Cr. showed comparative results in which the effects of SC.Cr. at both doses were superseded as compared to that of FC.Cr. (Figures 4(b), 4(C)).

### 3.5. FC.Cr. and SC.Cr. Ameliorate SOD and MDA

**Figure 5:**
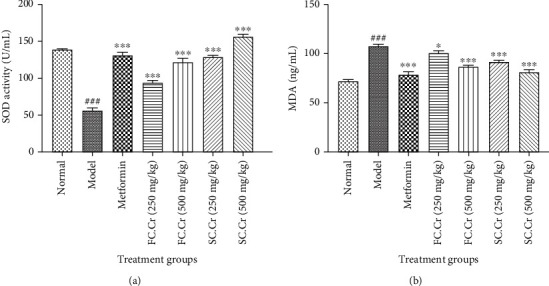
Effects of FC.Cr. and SC.Cr. on plasma levels of SOD (U/mL) and MDA (ng/mL) in HFD-STZ-induced rats. FC.Cr. and SC.Cr. were administered at 250 mg/kg and 500 mg/kg after three weeks till the end of study duration. SOD (a) and MDA (b) were measured after drawing plasma from the treated and normal group of rats. The values are expressed as the mean of six animals in a group. Statistical analysis is performed with one-way ANOVA followed by Tukey's test. All the groups are compared with the HFD-STZ group, while the HFD-STZ group is compared with the normal group. Results are considered significant (^∗^) if*P* < 0.05, (^∗∗^) and if*P* < 0.01and highly significant (^∗∗∗^) if*P* < 0.001.

The plasma level of SOD was significantly curtailed in the diabesity-induced group. The level was favorably brought to normal in a significant and dose-dependent manner with the administration of FC.Cr. and SC.Cr. (Figure 5(a)). FC.Cr. at 250 mg/kg and 500 mg/kg brought the SOD levels up to 96 ± 5 U/mL and 115 ± 5 U/mL, respectively. Similarly, SC.Cr. raised the level of SOD to 125 ± 5 U/mL and 155 ± 5 U/mL when administered at the doses of 250 mg/kg and 500 mg/kg, respectively. The dose comparison of both plants at equivalent doses shows the superior effects of SC.Cr. on SOD. Similarly, both FC.Cr. and SC.Cr. considerably reduced the raised levels of MDA at 250 mg/kg and 500 mg/kg (Figure 5(b)). It was noticed that the effects of SC.Cr. at both comparative doses surpassed the FC.Cr. effects.

## 4. Discussion

Diabesity has been turned into a major clinical issue all over the globe as a result of modern lifestyle adaptations and complex dietary habits. It is a term that has been coined to cover the interface of obesity and diabetes. Data in the past two decades have revealed that obesity is one of the major risk factors of diabetes [38, 39]. The presence of diabetes in obese individuals complicates the overall metabolic scenario in the body that has a potential to develop into metabolic syndrome and cardiovascular complications [40, 41]. As most of the scientific efforts are concentrated towards finding an isolated treatment for diabetes or obesity, only a handful of studies highlight the combined treatment options for both diabetes and obesity. However, in modern times, diabesity has achieved a significant attention from medical professionals and researchers, owing to its increasing prevalence worldwide. The prevalence of diabesity is drastically affecting the working efficiency and lifestyle of the obese people.

The herbal method of treatment remains a cornerstone of therapeutics. Medicinal plants have been used for centuries to treat diabetes and obesity. This study aimed to use two medicinally active plants in diabesity with a significantly established metabolic profile. The plants were selected through a literature survey and evaluation of their potential as pharmacological agents. The FC.Cr. fruit is rich in sugars, minerals, phenolic mixtures, and vitamins. It has high contents of polyphenols and amino acids. Its components are free of fat and cholesterol [42]. Similarly, the methanolic extract of seeds of SC.Cr. has different phytochemicals which are effective in different diseases. The presence of glycosides, flavonoids, and tannins with alkaloids helps lower the blood glucose level. Similarly, saponins are reported to decrease cholesterol [43].

Diabesity animal models are considered as a useful method for the investigation of newer antidiabetic drugs. As human diabetic clinical conditions involve the features of insulin resistance, *β*-cell deficiency, and consistent hyperglycemia, the diabesity models seem to serve the purpose. STZ, 2-deoxy-2-(3-(methyl-3-nitrosoureido)-D-glucopyranose) is known to induce type 1 and T2DM in rats [44]. Recently, HFD/STZ-induced rodent diabetic models have gain popularity due to their relative ability to mimic the state of obesity along with diabetes depending upon the length of HFD administration [30]. Usually, 8 weeks of HFD/STZ model produce significant symptoms of hyperglycemia and hyperlipidemia [45]. Keeping in mind all these factors, we selected the HFD/STZ-induced model for 8 weeks in rats with slight modifications [46]. In our study, the diabesity model was successfully established that exhibited a significant increase in fasting blood glucose levels compared to normal rats which signifies insulin resistance/insufficient insulin release. Similarly, body weights were also monitored during study duration, which was found to notably increase in the HFD/STZ group. However, we noticed that the even significant increase in body weights of the HFD/STZ group was far lesser in terms of the number of grams increase where alone HFD was administered for a similar duration [47]. This can be due to the fact that STZ curbs the increase in body weights generally as recorded in previous studies [48, 49].

The pharmacological intervention with FC.Cr. and SC.Cr. in HFD/STZ-induced diabesity remarkably improved the morbid state. Both FC.Cr. and SC.Cr. reduced the increase in body weights, but a reduction was more pronounced with SC.Cr. as compared to FC.Cr. which even showed a nonsignificant decrease in body weights at 250 mg/kg of dose. Similarly, FBG was also curtailed by both extracts, but the extent of reduction of SC.Cr. surpassed FC.Cr. The decrease in body weights along with FBG the decline encouraged us to analyze the effects of FC.Cr. and SC.Cr. on lipid profile. It was noted that both FC.Cr. and SC.Cr. extraordinarily improve the HFD/STZ dysregulated plasma lipid profile. Despite the fact that both showed significant results, yet SC.Cr. exhibited a superior improvement in lipid profile as compared to FC.Cr. Further, we compared the finding of FC.Cr. and SC.Cr. at 250 mg/kg and found that the latter was more effective in lowering LDL, VLDL, TG, and cholesterol even at a lower dose. The pattern of plasma lipid control was more pronounced at 500 mg/kg in favor of SC.Cr.

Oxidative stress plays a vital role in both diabetes and obesity [50–52]. It is also well established that the raised oxidative stress is associated with increasing the risk factors of diabetes such as altering pancreatic insulin secretion and the effects of the hormone on target cells [53]. SOD is a major antioxidative enzyme which plays a pivotal role in the neutralization of reactive oxygen species (ROS) [54, 55] and lowers oxidative stress. We have found that both FC.Cr. and SC.Cr. increase the level of SOD in plasma. Similarly, MDA are the product of lipid peroxidation [56]. The effects of SC.Cr on SOD and MDA at both 250 mg/kg and 500 mg/kg were more significant as compared to FC.Cr. at both doses. Previous studies have shown that the concentration of MDA is increased in diabetes mellitus [57]. Early changes of collagen by sugar adducts that form a series of glycation products incite the degradation of the lipids to MDA and hence further cross-linking by MDA of the already modified collagen. We found that both FC.Cr. and SC.Cr. decrease the MDA to a considerable extent while the effects of SC.Cr. remained superior to FC.Cr.

It can be concluded that both FC.Cr. and SC.Cr. are effective in obesity-linked diabetes by regulating the various glucose, lipid metabolic products, and oxidative stress factors. The effects of SC.Cr. are found to be more prominent in all measured parameters. Further studies are required to clarify the exact mechanism of both plants.

## Figures and Tables

**Table 1:** Complete blood count (CBC) of the experimental animals.

Groups	Red blood cells (10^6/^UL)	White blood cells (10^3^/UL)	Platelets (10^3^/UL)	Hemoglobin (g/dL)
Normal	7.5 ± 0.25	7.0 ± 0.086^∗∗∗^	942 ± 11	13 ± 0.26
HFD-STZ	7.9 ± 0.15	13 ± 0.21	740 ± 54	13 ± 0.10
Metformin (500 mg/kg)	7.3 ± 0.19	11 ± 0.61	942 ± 68	13 ± 0.067
FC.Cr. (250 mg/kg)	8.4 ± 0.14	8.9 ± 0.66^∗∗∗^	850 ± 27	13 ± 0.088
FC.Cr. (500 mg/kg)	8.3 ± 0.11	8.1 ± 0.53^∗∗∗^	882 ± 98	12 ± 0.50
SC.Cr. (250 mg/kg)	8.2 ± 0.20	11 ± 0.34^∗∗^	879 ± 46	13 ± 0.10
SC.Cr. (500 mg/kg)	8.0 ± 0.49	8.0 ± 0.38^∗∗∗^	822 ± 107	13 ± 0.25

Data values are shown as mean ± SEM of 6 animals in each group. The results are evaluated by using the one way ANOVA then compared with the HFD-STZ group. The results are significant is*P* < 0.05(^∗^), more significant (^∗∗^) if*P* < 0.01, and highly significant (^∗∗∗^) if*P* < 0.001.

## Data Availability

The data used to support the findings of this study are available from the corresponding author upon request.
